# Differentiating cognitive or motor dimensions associated with the perception of fall-related self-efficacy in Parkinson’s disease

**DOI:** 10.1038/s41531-018-0059-z

**Published:** 2018-08-20

**Authors:** Taylor Chomiak, Alexander Watts, Jacqueline Burt, Richard Camicioli, Sun Nee Tan, Martin J. McKeown, Bin Hu

**Affiliations:** 1 Division of Translational Neuroscience, Department of Clinical Neurosciences, Hotchkiss Brain Institute, Alberta Children’s Hospital Research Institute, Cumming School of Medicine, University of Calgary, Calgary, AB T2N 4N1 Canada; 2 Department of Medicine, Division of Neurology, Clinical Sciences Building, University of Alberta, Edmonton, AB T6G 2R7 Canada; 3 Faculty of Medicine, University of British Columbia, Vancouver, BC V6T 1Z3 Canada

## Abstract

In Parkinson’s disease (PD), concurrent declines in cognitive and motor domain function can severely limit an individual’s ability to conduct daily tasks. Current diagnostic methods, however, lack precision in differentiating domain-specific contributions of cognitive or motor impairments based on a patients’ clinical manifestation. Fear of falling (FOF) is a common clinical manifestation among the elderly, in which both cognitive and motor impairments can lead to significant barriers to a patients’ physical and social activities. The present study evaluated whether a set of analytical and machine-learning approaches could be used to help delineate boundary conditions and separate cognitive and motor contributions to a patient’s own perception of self-efficacy and FOF. Cognitive and motor clinical scores, in conjunction with FOF, were collected from 57 Parkinson’s patients during a multi-center rehabilitation intervention trial. Statistical methodology was used to extract a subset of uncorrelated cognitive and motor components associated with cognitive and motor predictors, which were then used to independently identify and visualize cognitive and motor dimensions associated with FOF. We found that a central cognitive process, extracted from tests of executive, attentional, and visuoperceptive function, was a unique and significant independent cognitive predictor of FOF in PD. In addition, we provide evidence that the approaches described here may be used to computationally discern specific types of FOF based on separable cognitive or motor models. Our results are consistent with a contemporary model that the deterioration of a central cognitive mechanism that modulates self-efficacy also plays a critical role in FOF in PD.

## Fear of falling: Determining the contributing factors

A Canadian study shows that motor and cognitive impairments in patients with Parkinson’s disease (PD) can independently contribute to fear of falling (FOF). Falls are an important complication in PD and FOF may lead to a self-induced restriction of activity that can greatly decrease a patient’s quality of life. Taylor Chomiak at the University of Calgary and colleagues have used a machine learning approach to investigate potential predictors of FOF in 57 patients with mild to moderate PD. They found that despite a lack of significant motor symptoms, FOF may emerge from aberrant coordinated regulation of executive systems. Understanding whether a patient’s FOF is mostly associated with their degree of motor impairment or a deterioration of cognitive mechanisms that lead to an underestimation of their own motor abilities will help tailor the initial therapeutic management of FOF.

## Introduction

A common feature of progressive neurodegenerative diseases, such as Parkinson’s disease (PD), is that clinical manifestations often involve degenerative pathology in multi-systems in which motor and cognitive dysfunction can co-exist and interact to complicate efforts in symptom diagnosis and intervention.^1–3^ In PD, which carries one of the highest fall risk among neurological illnesses,^4^ both cognitive and motor-function decline can affect an individual’s ability to conduct daily tasks.^1,5,6^ For example, fear of falling (FOF), a function of one’s perceived risk of falling and cognitive functioning, is a common concern among the elderly and poses a significant barrier to physical and social activities, which can lead to a downward spiral in general health.^1^ FOF is significantly associated with actual falls, which together, may lead to a self-induced restriction of activity, reductions in muscle strength, and general physical de-conditioning that may serve to further increase fall risk.^1,7,8^ Importantly, FOF in and of itself is a significant determinant of health-related quality of life even more so than balance impairments or actual falling.^9–11^ However, although motor impairment and poor gait function are associated with FOF,^9,12–15^ individuals with PD can have obvious motor impairment and experience falls, but lack FOF.^16^ It would therefore be highly desirable to develop precision medicine methods that would be able to differentiate domain-specific contributions of cognitive or motor impairments with respect to FOF.

Previous research has used the Falls Efficacy Scale-International (FES-I) as a composite measure for fall concern and a state of FOF.^15,17^ Since the FES-I-defined state of FOF incorporates measures related to balance, previous falls, and depressive symptoms,^17^ the association between motor impairments and FOF is hardly surprising. Indeed, it has been frequently proposed that the therapeutic management of FOF should focus on targeting lower-level mobility problems.^1,9,12–14^ In addition to motor impairment, it is also well established that individuals with PD can have cognitive dysfunction early in the course of the disease that can occur independent of motor system pathology.^18,19^ FOF in PD therefore, may not necessarily emerge because of underlying motor system pathology, but rather because of poor cognitive functioning. In this context, deteriorating higher-level cognitive functioning may result in an inaccurate appraisal and underestimation of ones’ actual motor capabilities,^20^ thus resulting in increased uncertainty around fall-related self-efficacy and higher FES-I scores and FOF. Moreover, unlike a motor model of FOF, a cognitive dysfunction model of FOF would also predict that certain individuals with PD may actually overestimate their own physical efficacy.^21^ This is clinically important as it may lead to a lower level of caution and a greater likelihood of engaging in high fall-risk behaviors in these individuals.

Prior studies on the relationship of FOF and cognitive functioning in PD have yielded contradicting results, with some studies suggesting that there is a relationship between cognitive functioning and FOF, and others reporting that there is not.^13,16,22–24^ A notable feature of these studies is that predictor variable correlations and regressions are often conducted on similar domains or aspects of the same domain that are embedded within multiple clinical evaluations (different measures of gait, clinical scores, etc.). When interrelated predictor variables are correlated in regression models they can reflect a common or shared underlying dimension (i.e., variable),^13,25,26^ which can sometimes lead to difficulty in determining the contributions of individual predictors among a group of interrelated predictors. One approach to overcome this issue is to first separate clinical data into uncorrelated dimensions before predictive statistical analysis. In this study, we therefore used an approach that allowed us to extract uncorrelated measures of cognitive and motor function, which were then used to independently identify and visualize cognitive and motor dimensions associated with FOF.

## Results

### Extraction of cognitive and motor dimensions

Demographic and clinical data indicated that our patient cohort tended to be male, educated, and moderately impaired (Table 1). To reduce noise in the data and extract underlying features that are most sensitive to FOF, a dimensionality reduction was performed through principal component analysis (PCA). The collected test scores were first adjusted for age, sex, disease duration, and education, and then subjected to PCA to extract uncorrelated components that share underlying variance.^25^ We found that unlike unadjusted test scores which resulted in partial or mixed-domain components, adjusted scores yielded two separable domain-specific components related to motor and cognitive function. Hoehn & Yahr, UPDRS-III, and gait speed all loaded together onto one component (motor domain), while visuospatial, attention, and memory retrieval test scores all loaded together onto another component (cognition domain) (Table 2 and Supplementary Fig. S1). These domain-specific components represent the largest components, which together, account for over half of the total variance of the six variables used in the dimensionality reduction analysis (Table 2).

**Table 1:** Participant characteristics

Total sample, *n* = 57	
Age (yrs)	64.9 (9.1)
Disease duration (yrs)	5.8 (4.7)
Sex (men/women) (%)	62/38
Years of education (yrs)	15.4 (2.3)
Serial subtract 7^a^	3 (2–3)
Visuospatial^a^	5 (0–5)
Delayed word recall^a^	4 (0–5)
Gait speed (m/min)	68.6 (12.1)
UPDRS-III	18.5 (9.9)
Hoehn & Yahr^a^	2 (1–3)
FES-I score	22.3 (6.4)
FOF (no/yes) (%)	81/19

FOF (%) is based on a FES-I score of ≥28^15,17^

^a^Median (range)

**Table 2:** Components and component loadings^a^ from PCA^b^

Variable	Component 1 (cognitive)	Component 2 (motor)
Serial subtract 7	**0.789**	0.133
Visuospatial	**0.783**	0.135
Delayed word recall	**0.651**	0.133
Gait speed	0.223	**0.565**
UPDRS-III	0.181	**0.709**
Hoehn & Yahr	−0.022	**0.791**
Total variance (%)	29.05	24.92
Cumulative variance (%)	29.05	53.97

^a^Principal component loadings represent the correlation between the individual variable and each component (after rotation); bold values indicate component loadings with an absolute value >0.40

^b^Standardized residuals from the linear regression of the raw test scores adjusted for age, education, disease duration, and sex were used for PCA

### Association of cognitive and motor dimensions with FOF

Next, each individual’s component scores were computed and evaluated. Extracted component scores were used to construct logistic regression models to evaluate the relationship between FOF and the extracted domains. As can be seen in Supplementary Fig. S2, a single predictor model including the motor domain illustrates that decreasing motor component scores are associated with an increasing probability of FOF. The cognitive component demonstrated a similar pattern to that of the motor domain with respect to FOF in a single predictor model (Supplementary Fig. S2). However, the individual regression models do not allow us to evaluate the independent contribution of the cognitive domain. For this, we next entered the motor and cognitive component scores into a single logistic regression model. This analysis revealed that while decreases in both motor and cognitive domain functioning are associated with increased probability of FOF, the extracted cognitive component was a significant and independent predictor of FOF (Table 3). The odds ratios and 95% confidence intervals (CI) from the final logistic regression model are shown in Table 3.

**Table 3:** Logistic regression model

Variable	OR	95% CI	*p*-value
Motor dimension	0.334	0.141–0.791	0.013^a^
Cognitive dimension	0.330	0.144–0.752	0.008^a^
Interaction	0.587	0.315–1.095	0.094

^a^*p* < 0.05

### Identifying FOF boundaries extending along cognitive and motor dimensions

**Fig. 1:**
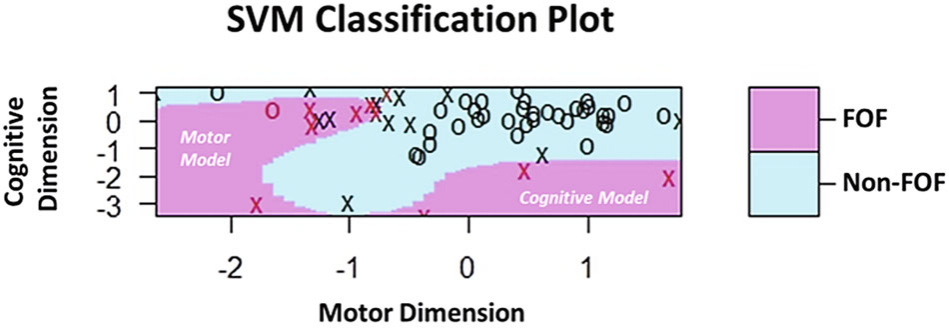
SVM classification plot based on cognitive and motor dimension scores. Purple and blue areas represent SVM defined FOF and non-FOF classification regions based on combined cognitive and motor dimension scores, respectively. “X” and “O” represent data from individuals, while the “X” denotes support vector data for model building. Red and black “X” and “O” coloring denotes FOF and non-FOF, respectively. Here, overall classification accuracy was greater than 92%. Note the model also identified boundaries between FOF; a region corresponding to poor cognitive functioning with relatively mild motor impairment (labeled as “Cognitive Model”), and a region represented by poor motor functioning (labeled as “Motor Model”) that also extends down along the cognitive dimension

As it is difficult for a single assessment tool, such as the FES-I to capture the underlying contributing factors of FOF, we wanted to examine if it was possible to identify FOF margins along cognitive and motor dimensions. This is important as it may help to uncover the dimensions that may be contributing to an individual’s FOF. For this we used the machine-learning algorithm support vector machines (SVM), which is an effective tool for classifying and defining variable boundaries along dimensions as the algorithm is designed to use a subset of the observed data to identify boundary conditions (i.e., the support vectors) for model building.^27,28^ Indeed, SVM not only revealed that there are boundaries and regions between FOF and non-FOF based on extracted cognitive and motor dimension scores (Fig. 1; purple vs. blue), but it also delineated boundaries that exist between FOF areas. The statistically distinct models resulting from SVM analysis included a cognitive model representing poor cognitive functioning with relatively mild motor impairment, and a motor model characterized by poor motor functioning with or without poor cognitive functioning (see Discussion and Fig. 1 for additional details).

## Discussion

In the current study we performed a new set of statistical procedures with the aim to more precisely identify the underlying features that are most sensitive to FOF. We found through dimensionality reduction that a cognitive domain component can serve as an independent predictor of FOF in PD. This unique cognitive domain, defined by visuospatial, attention, and memory functioning, may reflect a central cognitive mechanism (see below). The significant association between cognitive dysfunction and FOF persisted even after adjusting for impairments in the motor dimension, indicating that both motor and cognitive models of FOF may play an important, yet different, role in FOF.

Previous bivariate and multivariate analysis related to the topic of cognitive functioning and FOF in PD have yielded conflicting results.^13,16,22–24^ In particular, executive-related functioning has not been explicitly identified in these studies as an independent predictor of FOF in PD. In addition to confirming previous research identifying a motor model of FOF, unique to this study, we used statistical methodology and dimensionality reduction and identified a cognitive domain-specific component associated with FOF in PD based on standardized clinical test scores. This makes comparisons between domains immediately interpretable despite the different methods and scales used to assess them. The approaches described here can be used to computationally discern cognitive and motor models of FOF in PD. Given that our study sample represents individuals with fairly mild to moderate PD, the analytical and machine-learning approaches described here may help in the initial therapeutic management of FOF and the development of individualized treatment plans to facilitate precision medicine in PD.

The PCA method used here also produced an additional interesting result, the finding that visuospatial/executive skills (alternating trail making; visuoconstructional-cube; visuoconstructional-clock) and attention (serial subtract 7) both loaded highly onto the same component (Table 2 and Supplementary Fig. S1). This supports previous work suggesting that the frontal-mediated attention/working memory task of serial subtract 7 may also have internal visuospatial scratchpad and spatial components.^29^ In fact, previous work has suggested that “storage-plus-processing” tasks are linked to executive function by a shared underlying cognitive ability, which has been referred to in the literature as executive attention or central executive.^26,30^ This underlying dimension, defined as the higher-level neural component required to coordinate complex cognition, involves directing attention, maintaining task goals, decision making, and memory retrieval,^26,30^ all of which are required for the cognitive tasks used in the current study. This suggests that despite a lack of significant motor impairment, FOF may indeed emerge in the cognitive model from dysfunction of a more complex cognitive mechanism and that the independent contribution of this higher-level cognitive component, rather than the more specific cognitive domains of attention, visuospatial, and memory retrieval per se, may represent aberrant coordinated regulation of executive systems.

Clinically, dysfunction of complex cognitive control may take the form of disrupted ‘cognitive’ estimates of probability and cost, and an underestimate of functional abilities as a result of a dissociation between heightened subjective feelings of threat (i.e., fall-related self-efficacy), and objectively accurate cognitive threat calculations.^20,21,31^ However, as the cognitive neuropathological load increases, awareness of more severe motor deficits can decline which can lead to the opposite effect, an overestimate of functional abilities.^32,33^ This may also help explain the downward left-protruding non-FOF region in Fig. 1, and represent individuals that may be at a higher risk of falling (i.e., individuals with greater motor impairment, but fail to recognize/appreciate this). This interpretation is consistent with that of Mak et al. (2014), who reported that greater disease severity and cognitive impairment is not necessarily associated with increased FOF, despite increased fall risk.^24^

In the SVM analysis, we also identified an interesting non-FOF region directly above the motor model (Fig. 1). This suggests, albeit indirectly, that under the condition of intact cognitive functioning, FOF may not become a dominant clinical manifestation. This may be due to one’s continued ability for increased attentional focus and awareness of self-efficacy, and the conscious use of protective measures, such as cautious gait to provide a sufficient level of fall-related self-efficacy.^34,35^ This also raises the possibility that cognitive training may be an effective therapeutic approach to reduce FOF.

Prior work has demonstrated a relationship between self-efficacy and behavioral measures of cognitive performance, and that self-efficacy can be enhanced by the provision of efficacy information that reflects mastery.^36–38^ Therefore, self-efficacy, and thus FOF, is a modifiable psychological construct.^39^ This is important as it suggests that the therapeutic management of FOF may be better served by focussing efforts on appropriate targets; lower-order mobility issues with the motor model, and cognitive functioning targets for the cognitive model. With easily amenable App-based approaches for cognitive training,^40^ and the capacity for smartphone Apps to facilitate behavior change, recent advancements in mobile device technology^41–43^ make integration of smartphone technology into behavioral healthcare quite feasible even in the cognitive dimension.^44–46^

We do acknowledge the limitations of this study. The primary limitation of this study is its cross-sectional design. Our study sample therefore represents a static picture of individuals with fairly mild to moderate PD, many without FOF. A long-term longitudinal study would allow for a better predictive model to capture changes in cognitive and motor domains and the time-dependent transition to FOF within the same study sample. A second limitation is that we do not have fall history data. This is related to the fact that the individuals anticipated to report a fall history would also have greater cognitive dysfunction,^47,48^ and thus clearly limits the reliability of retrospective data like that typically collected for fall history.^49^ Future studies should therefore consider a prospective account of fall events to identify fall risk. Third, while an advantage of SVM is that it only considers instances that are close to the boundary; meaning that SVM is unaffected by instances far away from the boundary despite the fact that they may be greater in number,^27,28^ there is still a need for future work to verify these proof-of-concept findings in a larger and more heterogeneous PD population (i.e., mild to severe PD). This would allow for both, more robust boundary definitions as there would be a greater proportion of individuals with and without FOF at the boundary that could be used to define the boundary (i.e., support vectors), as well as further validating the localization of individuals to particular FOF models. Fourth, another limitation of the current study is that individuals were not evaluated on measures of depression and/or anxiety. This may be particularly relevant as cognitive dysfunction associated with FOF may also be driving certain neuropsychiatric aspects of PD such as increased anxiety.^50^ Finally, it is also worth mentioning that extracted cognitive and motor domain scores were not adjusted for l-Dopa equivalent daily dose. However, similar to previous work,^25^ extracted scores were instead adjusted for disease duration, which is significantly associated with l-Dopa equivalent daily dose even after controlling for other demographic and disease-specific measures.^51,52^ Strengths of this study on the other hand are that it incorporated data from multiple centers, and that it used the FES-I to conceptualize FOF. Despite the fact that the individuals in the current study did not undergo comprehensive neuropsychiatric testing, and with the limitations of our cross-sectional design, our use of FES-I-defined FOF has the added advantage of using a FOF conceptualization, which incorporates measures of balance and neuropsychiatric symptoms through which the convergent and predictive validity has been explored extensively in a longitudinal design.^17^

To conclude, as the nature of FOF is multi-factorial, it is difficult for a single assessment tool, such as the FES-I to capture the underlying contributing factors to FOF. Simply identifying FOF in PD may not be sufficient. The approach presented here, on the other hand, may not only be able to identify PD individuals with FOF, but may also help identify the underlying model contributing to FOF. This form of precision medicine may ultimately have a transformative effect on the therapeutic management of FOF in PD.

## Methods

### Participants

This is a cross-sectional observational study that included a convenience sample of 57 (*n* = 57) individuals with PD from an ongoing registered intervention trial (ISRCTN06023392). Ethics approval from the University Ethics Board for Human Research (REB13-0009) and informed written consent were obtained. Our sample size was guided by a recommendation of having approximately 10 times as many participants as dimensionality reduction variables,^53^ which was to be subsequently verified by the Kaiser–Meyer–Olkin measure of sampling adequacy. All PD participants were initially recruited via clinician referral by a neurologist at the University of Calgary, University of Alberta, and University of British Columbia, and through Parkinson Alberta Society support groups. These patients had a confirmed diagnosis of PD, were on a steady medication routine, and were tested in the “on” state.

### Clinical testing

The participants completed Unified Parkinson’s Disease Rating Scale Part 3 (UPDRS-III) and Hoehn & Yahr staging by a trained practitioner. For cognitive executive function tests, we used standard visuospatial function (alternating trail making; visuoconstructional-cube; visuoconstructional-clock), attention processing (serial subtract 7), and short-term memory retrieval (delayed word recall).^54^ These cognitive tasks collectively test one’s storage-plus-processing capabilities, which are thought to rely on a common underlying cognitive process.^26,30^ Fall-related self-efficacy and FOF was assessed by the 16-item FES-I, which scores the level of concern about falls for a range of activities of daily living related to social and physical activities inside and outside the home,^55^ and has been clinically validated for PD.^56^ FOF is conceptualized by a “high-degree of fall concern” defined by a cut-off value determined by the state variables of balance, previous falls, and depressive symptoms.^17^ The state of FOF, therefore, is defined by a total FES-I score of ≥28.^15,17^

### Walking test

Self-selected walking speed, or gait velocity provides a quantifiable index of ambulatory ability and gait functioning.^57^ To obtain an objective and quantitative assessment of walking speed, patients wore a wearable sensor (Ambulosono Sensor System^58,59^) and were instructed to walk in a defined area on-site at a self-selected pace. The walking time was controlled by Ambulosono, which runs off the iOS GaitReminder App that can issue auditory instructions while continuously recording step size via iOS gyro and accelerometers (after corrections for limb length, angular excursion, signal filtering, and drift).^60^ Step parameters obtained via GaitReminder have an average of <10% difference when tested against direct video measurements, and a similar error rate was also found when the App was used for treadmill walking or over-ground ambulation where actual values can be obtained from direct machine reading or physical measurement (e.g., markings on the floor).^58,61^ Similar results have also been obtained in a subsequent cohort (not shown).

### Data and statistical analysis

All clinical and demographic data from our sample were included in our analysis. Descriptive statistics were calculated for demographic, clinical, and cognitive test scores (Table 1). Hoehn & Yahr and UPDRS-III were transformed into the same direction (i.e., assessment possible maximum score minus actual score). This means subjects that score lower (i.e., less motor impairment/disease severity) will have a higher transformed score. To avoid redundancy and account for underlying correlation patterns, a dimensionality reduction was performed through PCA. Linear regression was used to adjust raw test scores for age, disease duration, years of education, and sex, and the standardized residuals for each of the six predictor variables were included in the PCA. PCA components were extracted with an eigenvalue threshold of 1 or greater, and the components were rotated using a varimax rotation.^25^ The Kaiser–Meyer–Olkin measure verified sampling adequacy (KMO > 0.5). Component scores were then used in logistic regression models. SVM modeling^27,28^ was used to visualize FOF boundaries along extracted cognitive and motor dimensions. For our model hyperparameters we used a soft-margin constant *C* = 10 for the decision boundary and gamma = 0.5 to prevent over-fitting (verified with cross-validation). A radial basis function kernel was used. All analyses were conducted using IBM SPSS Statistics and R. Tests of significance were computed via the Wald statistic, and *α* was set at 0.05.

### Data availability

The data that support the findings of this study are available from the corresponding authors upon reasonable request.

### Code availability

R (R Core Team (2017). R: A language and environment for statistical computing. R Foundation for Statistical Computing, Vienna, Austria) and package e1071 are freely available (https://www.R-project.org/).

## Electronic supplementary material

Supplementary Material

## Electronic supplementary material

**Supplementary information** accompanies the paper on the *npj Parkinson’s Disease* website (10.1038/s41531-018-0059-z).
