# Supplementary material for: Differentiating cognitive or motor dimensions associated with the perception of fall-related self-efficacy in Parkinson’s disease
Source: NPJ Parkinsons Dis. 2018 Aug 20;4:26. doi: 10.1038/s41531-018-0059-z (PMC6102294; doi:10.1038/s41531-018-0059-z)
Supplement: Supplementary file 1 — Supplementary Material [file 41531_2018_59_MOESM1_ESM.pdf]

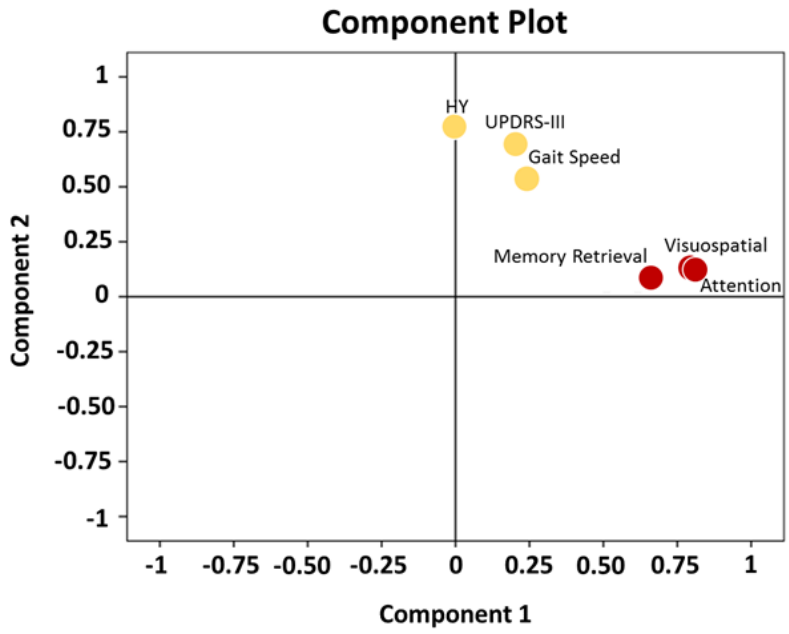

Supplementary Figure S1. Principal component loadings for motor and cognitive clinical test scores

Principal component loadings represent the correlation between the individual variable and each component after rotation (also see Table 2). Cognitive and motor domain components are shown in red and yellow, respectively. Components are based on the standardized residuals from the linear regression of the raw test scores adjusted for age, education, disease duration, and sex.

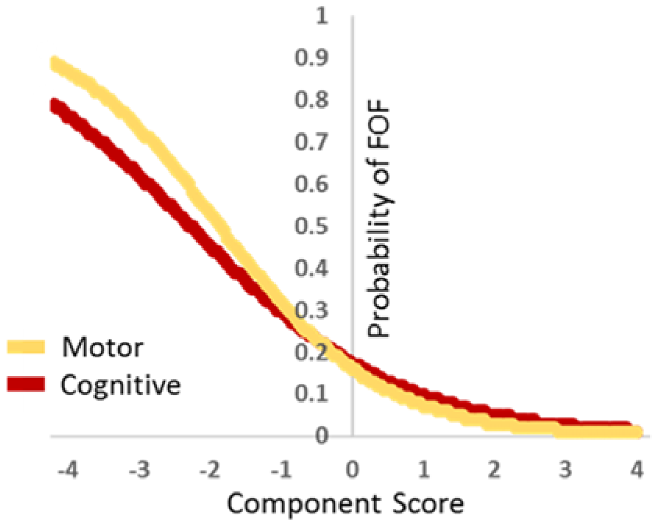

Supplementary Figure S2. Relationship between cognitive and motor domain component scores and probability of FOF

Extracted component scores from the dataset were used to construct logistic regression models to evaluate the estimated probability of an individual exhibiting FOF with respect to a given cognitive or motor domain score (red and yellow, respectively). Extracted component scores are based on standardized residuals (see Supplementary Fig. S1).
